# PRESIDENTIAL SYMPOSIUM: RACIAL AND ETHNIC HEALTH DISPARITIES ACROSS THE LONG-TERM SERVICES AND SUPPORTS CONTINUUM: NEED FOR POLICY AND ACTION

**DOI:** 10.1093/geroni/igac059.1197

**Published:** 2022-12-20

**Authors:** Debra Dobbs, Larry Polivka

## Abstract

The pandemic further exacerbated racial and ethnic health disparities with BIPOC (Black, Indigenous and People of Color) communities experiencing significantly higher infection rates and higher hospitalization rates compared to White populations. Underlying medical conditions that disproportionately affect BIPOC populations (e.g., cardiovascular disease, diabetes, COPD) and social determinants of health (income, community and environmental factors, access to health care and job security) can account in large part for increased rates of morbidity and mortality. Within the long term services and supports (LTSS) continuum, health disparities exist in both who receives and provides care. The four papers in this Social Research Policy and Practice Presidential Symposium will highlight factors that result in health disparities across the LTSS continuum and propose possible policy and practice solutions to address the disparities. The first paper presented will be an overview of existing nursing home workforce issues that continue to impact disparities in the quality of resident care. The second paper presented examines nursing home resident quality of life disparities from a measurement perspective. The third paper will highlight issues on elder abuse and mistreatment of rural elders, American Indian and Alaskan Natives. The fourth paper will present recommendations from key stakeholders of the RAISE Family Caregiving Advisory Council on how to meet the needs of underrepresented groups (AAPI, BIPOC, Indigenous peoples) of family caregivers. Policy and practice solutions to reduce disparities will be discussed.

